# Evaluation of IL-35, as a Possible Biomarker for Follow-Up after Therapy, in Chronic Human *Schistosoma* Infection

**DOI:** 10.3390/vaccines11050995

**Published:** 2023-05-17

**Authors:** Nadia Marascio, Maria Teresa Loria, Grazia Pavia, Cinzia Peronace, Neill James Adams, Morena Campolo, Francesca Divenuto, Angelo Giuseppe Lamberti, Aida Giancotti, Giorgio Settimo Barreca, Maria Mazzitelli, Enrico Maria Trecarichi, Carlo Torti, Francesca Perandin, Zeno Bisoffi, Angela Quirino, Giovanni Matera

**Affiliations:** 1 Clinical Microbiology Unit, Department of Health Sciences, “Magna Græcia” University of Catanzaro—“Mater Domini” Teaching Hospital, 88100 Catanzaro, Italy; 2 Infectious and Tropical Diseases Unit, Padua University Hospital, 35128 Padua, Italy; 3 Infectious and Tropical Diseases Unit, Department of Medical and Surgical Sciences, “Magna Graecia” University—“Mater Domini” Teaching Hospital, 88100 Catanzaro, Italy; 4 Department of Infectious, Tropical Diseases and Microbiology, IRCCS Sacro Cuore Don Calabria Hospital, Negrar di Valpolicella, 37024 Verona, Italy

**Keywords:** *Schistosoma haematobium*, *Schistosoma mansoni*, IL-35

## Abstract

The host response to helminth infections is characterized by systemic and tissue-related immune responses that play a crucial role in pathological diseases. Recently, experimental studies have highlighted the role of regulatory T (Tregs) and B (Bregs) cells with secreted cytokines as important markers in anti-schistosomiasis immunity. We investigated the serical levels of five cytokines (TNFα, IFN-γ, IL-4, IL-10 and IL-35) in pre- and post-treatment samples from chronic *Schistosoma* infected patients to identify potential serological markers during follow-up therapy. Interestingly, we highlighted an increased serum level of IL-35 in the pre-therapy samples (median 439 pg/mL for *Schistosoma haematobium* and 100.5 pg/mL for *Schistsoma mansoni* infected patients) compared to a control group (median 62 pg/mL and 58 pg/mL, respectively, *p* ≤ 0.05), and a significantly lower concentration in post-therapy samples (181 pg/mL for *S. haematobium* and 49.5 pg/mL for *S. mansoni* infected patients, *p* ≤ 0.05). The present study suggests the possible role of IL-35 as a novel serological biomarker in the evaluation of *Schistosoma* therapy follow-up.

## 1. Introduction

Schistosomiasis is a neglected tropical disease caused by parasitic trematodes of the genus *Schistosoma* affecting more than 230 million people worldwide, mainly in countries with tropical and subtropical climates [1,2]. Over the last few years, the increase in migration and international tourism has stimulated an increasing public health interest in schistosomiasis [3,4]. Six main species of *Schistosoma* are responsible for infection in humans: *S. mansoni*, *S. haematobium*, *S. japonucum*, *S. intercalatum*, *S. guineensis*, and *S. mekongi*, with *S. haematobium* and *S. mansoni*, being the most widespread. There have also been reports of human infection with hybrid species involving *S. haematobium*-*bovis* and *S. curassoni*-*matthei* (from cattle) in humans [5,6,7]. *Schistosoma* infection is often acquired during childhood due to frequent contact with infected freshwater and chronic illness usually develops in adults [6]. Complications can include severe liver and intestinal pathology, as well as urolithiasis, nephritis and bladder cancer [6]. Overall, three clinical stages of schistosomiasis can be described: (i) the first, called cercarial dermatitis, occurs 24 h after penetration of the cercariae into the dermis; (ii) the acute phase that appears 3–8 weeks after infection; and (iii) the chronic phase that occurs months or years after infection, with the formation of granulomas around the *Schistosoma* eggs trapped in tissue [3].

During the acute phase of infection, larval forms of the parasite trigger the host immune response to activate pro-inflammatory Th1 and anti-inflammatory Th2 pathways in turn, leading to recruitment and bone marrow release of granulocytes and mast cells, as well as synthesis and release of IgE. The adult form of *Schistosoma* has evolved mechanisms for evading and suppressing the host immune response, which enables it to survive in the host for many years, leading to chronic infection [8]. The exact phenotype of the induced response depends on a complex immunological “dialogue” involving cytokines and immune cells. During infection, the Th1/Th2 responses and the immune-modulating activities by regulatory T (Treg) and B (Breg) cells reflect the interaction between the host immune system and the development of the parasite inside the host, with Th1 (pro-inflammatory) responses (production of IFN-γ, TNFɑ, IL-6, and IL-1) prevailing in the initial acute phase, followed by a Th2 response stimulated by egg antigens and characterized by the production of IL-4, IL-5, IL-13 and IL-10 [9,10,11]. The Th2 response regulates Th1-mediated immunopathology and exerts anti-inflammatory effects. However, it has also been reported that this immune regulation can be initiated during the acute phase [12] following multiple exposure to *Schistosoma* cercariae. A decrease in the Th1 responses is enabled by an IL-10-dependent mechanism of immune modulation [13]. The helminth-induced type 2 immune responses may cause the formation of granulomas during schistosomiasis. The severity of intestinal schistosomiasis is related to recurrent exposure and to the number of parasite eggs present in the mucosa [3,14].

During chronic schistosomiasis, a general T cell hypo-responsiveness with downregulated antigen-specific Th1 and Th2 cell responses has been reported [15,16]. *Schistosoma* has developed several mechanisms to manipulate the regulatory cellular reservoir of the host to ensure the long-term persistence of the parasite in the host. Regulatory Treg and Breg cells have been shown to be involved in the induction and maintenance of T-cell anergy [17,18]. The proliferation of Treg and Breg during chronic infection is associated with suppressed development of pathology [19] and down-modulated Th1 and Th2 responses [20,21], promoting parasite survival within the host [22,23]. Treatment with the anti-schistosomal drug praziquantel (PZQ) leads to clearance of infection [24], elevated antigen-specific T-cell proliferation [15], and cytokine production [10,16]. However, after PZQ treatment, it an increase in effector T-cell frequencies (Th1 and Th2) with increased antigen-specific cytokine production was observed, with a concurrent decrease in the levels of the Treg subpopulation and their secreted specific cytokines [24].

Interleukin-35 (IL-35), a mediator of Breg cells, has been shown to be an important anti-inflammatory molecule, belonging to the heterodimeric IL-12 family of cytokines [25]. Indeed, it has yet been reported that IL-35 plays a relevant regulatory role in different clinical conditions, including rheumatoid arthritis, diabetes mellitus, Systemic Lupus Erythematosus, psoriasis and cancer [26]. However, the involvement of IL-35 during schistosomiasis has not yet been fully investigated, although the immunosuppressive mechanism of *Schistosoma* has been frequently reported during human infection [27]. Previous investigations regarding immunological responses to *Schistosoma* infections have been carried out using either in vitro cultures of human immune cells (e.g., PBMC, macrophages, or lymphocytes) or in vivo experimental models using animals or in vitro cultures of animal cells. Although these two models are appropriate for evaluating the role of single parasite products (e.g., *Schistosoma* egg antigen, SEA), the outcome of the *Schistosoma* infection is very difficult to extrapolate from such models. 

Herein, we analyze a range of cytokines representing the Th1, Th2, Treg, and Breg immune responses in serum samples of chronic *Schistosoma*-infected patients before and after anti-parasitic therapy to identify potential biomarkers in the evaluation of *Schistosoma* follow-up therapy.

## 2. Materials and Methods

### 2.1. Study Design and Population

The present investigation followed a retrospective observational design. Between December 2015 and August 2019, patients were enrolled by the Infectious and Tropical Diseases Unit of the University Hospital in Catanzaro and by the Department of Infectious-Tropical Diseases and Microbiology, IRCCS Sacro Cuore Don Calabria Hospital, Negrar di Valpolicella (Verona). A control group was chosen from healthy volunteers of the graduate student population at the Magna Graecia University in Catanzaro, Italy, as it was impossible to guarantee that the immigrant study population was parasite free, even following a negative parasitological exam.

Parasitological screening of stool and urine specimens by microscopy of the migrant population and control group was carried out. Fecal samples were processed following Ritchie’s concentration technique with formalin-ethyl acetate, while urine specimens were centrifuged, and the sediment analyzed. The TNFα, IFN-γ, IL-4, IL-10, and IL-35 cytokine quantification was carried out on serical samples from *Schistosoma* infected patients, collected before and after anti-parasitic therapy, alongside the control group of healthy volunteers. The time span from serical pre- and post-treatment samples varied between two and three weeks, based on the different time when patients returned to hospital for clinical control.

### 2.2. Measurement of Serum Cytokines

Serum samples were obtained from blood specimens collected before and after therapy and stored at −80 °C until processed. TNFα, IFN-γ, IL-4, and IL-10 were evaluated using the “Evidence Investigator” semiautomatic instrument with the “cytokine kit high sensitivity” panel (Randox Laboratories Ltd., Crumlin, UK) able to detect Th1, Th2, and Treg cytokines, chemokines, and growth factors. IL-35 was measured using ELISA with a human IL-35 kit (MyBioSource, San Diego, SoCal, CA, USA) in all cases according to the manufacturer’s protocol.

### 2.3. Statistical Analysis

All data are expressed as medians and quartile ranges. Statistical analysis was carried out using the Kruskal–Wallis test plus Dunn’s test, with a *p* value ≤ 0.05 considered statistically significant. All analyses were performed using GraphPad 8.0 (GraphPad Software Inc., San Diego, CA, USA).

## 3. Results

### 3.1. Study Cohort

Between December 2015 and August 2019, migrant patients were screened at the University Hospital of Catanzaro, Italy, and at the IRCCS Sacro Cuore Don Calabria Hospital, Negrar di Valpolicella (Verona), Italy. Among the migrant population, 35 subjects were positive for *Schistosoma* infection: *S. haematobium* (n = 15), *S. mansoni* only (n = 8), *S. mansoni* with other parasite co-infection (n = 8) and hybrid *S. haematobium-bovis* (n = 1). A control group of healthy volunteer subjects (n = 9) was included in the study. Demographic characteristics and microscopical examination data from biological samples of chronic *Schistosoma* infected patients are reported in Table 1.

In co-infected patients, all non-*Schistosoma* parasites were intestinal protozoa (*Entamoeba coli*, *Blastocystis hominis*, *Iodamoeba butschlii* and *Giardia intestinalis*). Praziquantel was administered at the dosage of a single 60 mg/kg in all cases, except in a pregnant woman (ID patient 11), to whom we administered two doses of 30 mg/kg three hours apart from each other. In patients who were diagnosed with other intestinal parasites, we prescribed metronidazole, mebendazole or albendazole, as per clinical indication [28,29,30].

After therapy, all patients treated were found to be free of eggs in urine and fecal samples.

### 3.2. Th1 Response

#### 3.2.1. TNFα Serical Levels before and after Anti-Parasitic Therapy

**Figure 1:**
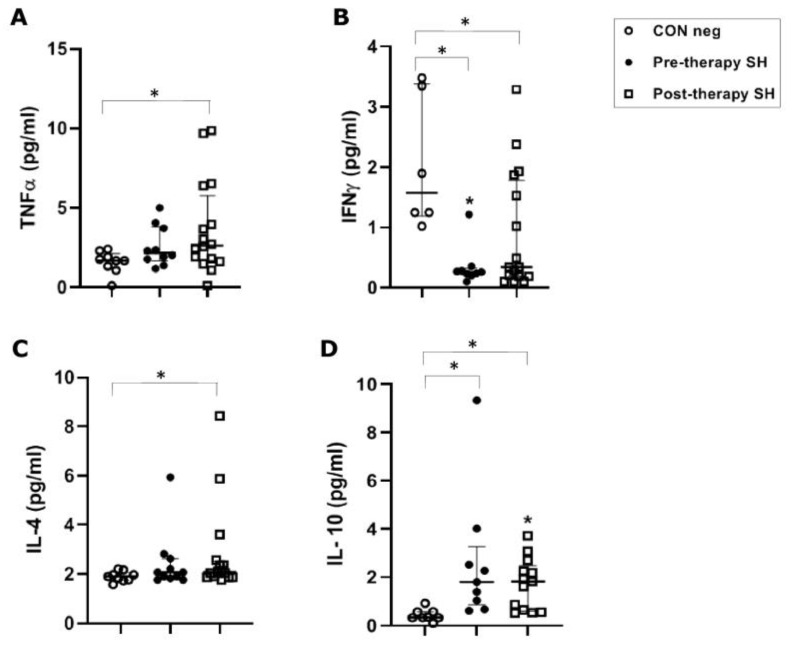
Cytokine concentrations in serum samples from pre-therapy (Pre-therapy SH) and post-therapy (Post-therapy SH) *Schistosoma haematobium* (SH) patients (n = 15) vs. healthy controls (CON Neg) (n = 9). Values are expressed as median and quartile ranges; significant differences were evaluated by the Kruskal–Wallis test plus Dunn’s test. (**A**) TNFα. * *p* ≤ 0.05 post-therapy vs. CON Neg; (**B**) IFNγ. * *p* ≤ 0.05 pre- and post-therapy vs. CON Neg; (**C**) IL-4. * *p* ≤ 0.05 post-therapy vs. CON Neg; (**D**) IL-10. * *p* ≤ 0.05 pre- and post-therapy vs. CON Neg.

In *S. haematobium*-infected patients, before anti-parasitic therapy, TNFα concentrations (median value 2.16 pg/mL) were not significantly increased when compared to healthy controls (1.68 pg/mL). Following PZQ therapy, TNFα was significantly increased vs. healthy control (*p* ≤ 0.05), but showed no significant increase vs. the pre-therapy group (Figure 1A).

**Figure 2:**
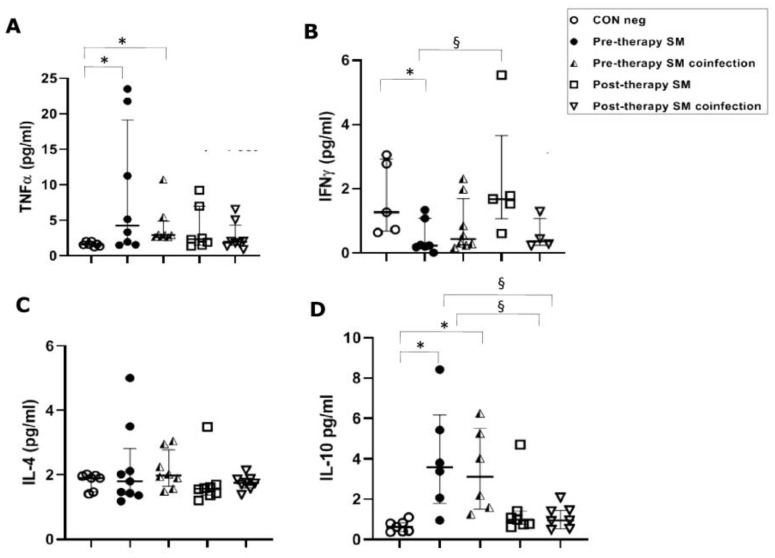
Cytokine concentrations in serum samples from pre-therapy (Pre-therapy SM) and post-therapy (Post-therapy SM) *Schistosoma mansoni* (SM) patients (mono- and co-infected) (n = 16) vs. healthy controls (CON Neg) (n = 9). Values are expressed as median and quartile ranges; significant differences were evaluated by the Kruskal–Wallis test plus Dunn’s test. (**A**) TNFα. * *p* ≤ 0.05 pre-therapy vs. healthy control (mono- and co-infected); (**B**) IFN-γ. * *p* ≤ 0.05 pre-therapy vs. control and § *p* ≤ 0.05 post therapy vs. pre-therapy (mono-infected only); (**C**) IL-4. No significant differences found; (**D**) IL-10. * *p* ≤ 0.05 pre-therapy vs. control and § *p* ≤ 0.05 post-therapy vs. pre-therapy.

However, in *S. mansoni*-infected patients, TNFα concentrations were significantly (*p* ≤ 0.05) higher in the pre-therapy group, both in single-infected patients (4.25 pg/mL) and in co-infected patients (2.93 pg/mL), with respect to concentrations found in healthy controls (1.60 pg/mL). TNFα concentrations did not show changes in post-therapy patients compared to the control group and pre-therapy sample values in patients with both single and co-infections (Figure 2A).

#### 3.2.2. IFN-γ Serical Levels before and after Anti-Parasitic Therapy

The *S. haematobium*-infected patients, before anti-parasitic therapy, showed a significant (*p* ≤ 0.05) decrease (0.260 pg/mL) of IFN-γ serical levels vs. the control group (1.57 pg/mL). Following therapy, there was a non-significant increase in concentration compared to the pre-therapy group, but the concentration remained significantly lower (0.34 pg/mL) than the control group (*p* ≤ 0.05) (Figure 1B).

In *S. mansoni* mono-infected patients, a significant (*p* ≤ 0.05) decrease in IFN-γ serical levels in the pre-therapy group (0.23 pg/mL) compared to the control group (1.27 pg/mL) was found. This was followed by a significant increase after PZQ therapy (1.68 pg/mL) compared to the pre-therapy group (*p* ≤ 0.05), but a non-significant increase compared to the control group. Surprisingly, these changes were not mirrored in the co-infection population, which showed neither a significant decrease in pre-therapy nor an increase in post-therapy compared to the control group (Figure 2B).

### 3.3. Th2 Response

#### IL-4 Serical Levels before and after Anti-Parasitic Therapy

In *S. haematobium*-infected patients, IL-4 concentrations (2.07 pg/mL) were not significantly different between pre-therapy and the control group (1.90 pg/mL). Following therapy, there was a significant (*p* = 0.05) increase compared to the control group. No significant difference was found comparing pre- and post-therapy groups (Figure 1C).

In *S. mansoni*-infected patients, we found no significant differences between the control group and the studied groups, such as mono- and co-infected or pre- and post-therapy (Figure 2C).

### 3.4. Treg Response

#### IL-10 Serical Levels before and after Anti-Parasitic Therapy

In *S. haematobium*-infected patients, IL-10 serum concentrations in pre- and post-therapy patients showed significantly (*p* ≤ 0.05) increased values (1.83 pg/mL) when compared to the control group (0.34 pg/mL) (Figure 1D). In the *S. mansoni*-infected patients, serical levels of IL-10 showed a significant (*p* < 0.05) increase (3.59 pg/mL of mono-infection vs. 3.11 pg/mL of co-infection) compared to the control group (0.63 pg/mL). Additionally, IL-10 also showed a significant (*p* ≤ 0.05) decrease after PZQ therapy compared to pre-therapy (1.04 pg/mL vs. 0.95 pg/mL, respectively) both in mono- and co-infected patients (Figure 2D).

### 3.5. Breg Response

#### IL-35 Serical Levels before and after Anti-Parasitic Therapy

**Figure 3:**
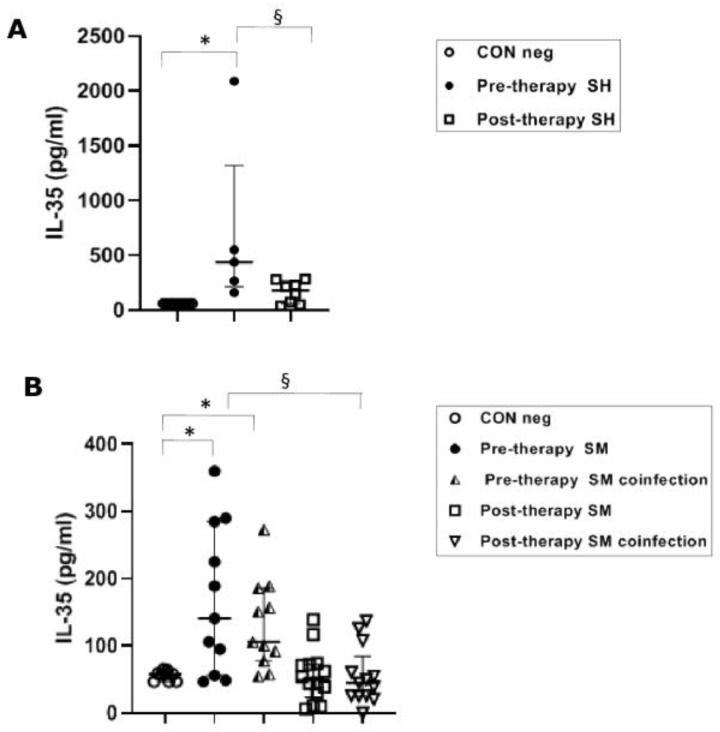
IL-35 cytokine concentrations in serum samples from *Schistosoma haematobium (SH)* (n = 15) and *Schistosoma mansoni (SM)* mono- and co-infected patients (n = 16) and healthy controls (CON neg) (n = 9) pre- and post-therapy. Values are expressed as median and quartile ranges; significant differences were evaluated by the Kruskal–Wallis test plus Dunn’s test. (**A**) * *p* ≤ 0.05 pre-therapy vs. control and § *p* ≤ 0.05 post-therapy v pre-therapy; (**B**) * *p* ≤ 0.05 pre-therapy vs. control (mono- and co-infected) and § *p* ≤ 0.05 post therapy vs. pre-therapy (mono- and co-infected).

Pre-therapy *S. haematobium*-infected patients revealed serical levels of IL-35 significantly (*p* ≤ 0.05) higher (439 pg/mL) than the control group (62 pg/mL). The post-therapy group showed IL-35 levels (181 pg/mL) significantly (*p* ≤0.05) lower than the pre-therapy group (Figure 3A).

The IL-35 levels in both mono-infected and co-infected groups during *S. mansoni* infection showed a significant (*p* ≤ 0.05) increase in the pre-therapy groups (100.5 pg/mL, mono-infected and 103.5 pg/mL, co-infected) compared to the control group (58 pg/mL). After PZQ therapy, we found a significant (*p* ≤ 0.05) decrease in both groups (49.5 pg/mL, mono-infected and 45 pg/mL, co-infected) compared to the pre-therapy mono- and co-infected groups (Figure 3B).

## 4. Discussion

In this study, we analyzed samples obtained from *Schistosoma* positive patients who had the natural progression of the illness. The cohort included young adults of migrant populations from countries endemic for schistosomiasis. Parasitological tests detected *Schistosoma* eggs in the feces or urine, indicating the presence of mature adult *Schistosoma*. No information was available regarding the time of infection, but since infection often dates to childhood, it can be assumed that our study population was all in the chronic phase of infection and very likely during the late regulatory phase of immune reaction.

Several studies have focused on the Th1 and Th2 cytokines [24,31,32,33]. In contrast, the behavior of cytokines from the Treg and Breg lymphocyte subsets during infections caused by the main *Schistosoma* species has not been fully investigated. The local microenvironment (e.g., cytokines and *Schistosoma* antigens) influences the plasticity of Th cells, which is regulated by various immune cells (e.g., macrophages, B cells, etc.) through a complicated network of interactions [34]. Infection with *Schistosoma* generally stimulates a Th1 response during the acute phase, with a consequent increase in pro-inflammatory cytokines TNFα and IFNγ, due to invasion by the larval cercaria, followed by Th1 suppression and adaptation to a Th2 response during the chronic phase of adult *Schistosoma* and egg release. This Th2 response leads to an increase in cytokines IL-4, IL-5, and IL-13, among others. However, the Th2 response is downregulated by the regulatory pathways Treg and Breg, causing an increase in IL-10 and IL-35. This allows the parasite to co-exist within the human host without leading to excess immune-related pathology [35]. The balance between Th1 and Th2 responses and their regulatory pathways has also been studied in other parasitic infections, such as leishmaniasis [36]. In vivo, these events are very likely non-sequential but occur simultaneously and are possibly related to parasite loads [35,37]. Labuda and colleagues, in their longitudinal study results, show that infection with *S. haematobium* is associated with antigen-specific T cell hypo-responsiveness, alterations of the T-cell memory pool, and increased levels of T reg cells [24].

The data in our study tend to conform to the general pattern outlined above, but with some minor differences, potentially relating to the two *Schistosoma* species and other intestinal parasites in the *S. mansoni* co-infected group. In our experience, we correlated the Th1/Th2 and Treg/Breg responses, comparing patients with a single or mixed infection vs. the control group. In relation to the Th1 cytokines, IFNγ was observed to be significantly reduced in both *S. haematobium* and *S. mansoni*-infected pre-therapy patients, reflecting the Th1 suppression model. Following therapy, a significant increase was observed in the *S. mansoni* mono-infected group, while the *S. haematobium* group showed a non-significant increase. In particular, the *S. mansoni* mixed-infection group showed no increase following therapy. This may indicate mechanisms unrelated to the *Schistosoma* infection in play. This increase in IFNɣ following therapy may represent the host immune system now “recognizing” the dead adult worm and reacting as if in the acute phase of infection, with stimulation of the Th1 pathway. It has been reported that patients with severe fibrosis have been associated with elevated TNFα, whereas low fibrosis patients showed high levels of IFNɣ [31,32]. Therefore, our TNFα and IFNγ data in pre-therapy patients might suggest an eventual progression to fibrosis in our patients. One of the reflections of this cytokine behavior may be periportal fibrosis and ureteral fibrosis/calcium precipitation [37,38]. In *S. mansoni* mixed infections, regarding IFNγ, the presence of other protozoan parasites would break the Th1 suppression model, thus explaining the different behavior of IFNγ in mixed infections. 

During *S. haematobium* infection, TNFα concentration increased in the pre-therapy group without any significance vs. the control group. However, in the post-therapy group, there was a significant increase in the cytokine compared to the control group. By contrast, a significant increase in TNFα was found in the *S. mansoni* pre-therapy group compared to the control group and a significant decrease post-therapy compared to the pre-therapy group. Based on our data, live *S. haematobium* was able to inhibit the increase in this Th1 cytokine which increased after the death of the parasite, thus reflecting the Th1 suppression model. Probably the pre-therapy increases of the Treg and Breg cytokines were not enough to inhibit the pre-therapy increase in TNFα. 

Regarding the Th2 response, IL-4 concentrations during *S. mansoni* infection were unaltered versus the control group (either in the pre- or post-therapy phase). In *S. haematobium* patients, the levels of IL-4 in the pre-treatment group were not distinguishable from healthy controls. Still, the IL-4 concentrations found in the post-treatment group were significantly increased versus both the control and the pre-therapy groups. These data confirm that the Th2 response is depressed during chronic infection. The presence of alive adult worms regulates the most specific cytokine cascade (Th2) associated with helminth eradication. After specific anti-parasitic chemotherapy, the IL-4 increased as the Th2 pathway was liberated from regulation. One possible mechanism for this has been proposed in an experimental mouse model [39], demonstrating that adult *Schistosoma* can release extracellular vesicles (EVs) containing miRNAs coding for transcriptional factors that interfere in primary T cell differentiation towards the Th2 pathway. Following therapy, no EVs are released. Schistosomiasis is associated with elevated levels of circulating regulatory T cells [40]. A significant decrease in circulating Treg cells following PZQ therapy in *S. mansoni*-infected males has also been reported [8]. Therefore, curative treatment with PZQ in patients with *Schistosoma* infection resulted in changes in the immune response. These changes are due to both worm removal and immune responses to the parasitic antigens of the dying worms. However, in the last few years, some studies have investigated the potential direct effect of the anti-helminthic drug on host immune cells. Additionally, the PZQ reducing effect on regulatory cells reported above, the drug also enhances T-cell proliferation by promoting the differentiation of type 1 regulatory T-cells (Tr1 cells) that mainly produce IL-10, associated with the suppression of tissue inflammation. Thus, there is a possibility that PZQ itself induces Th1 cells to differentiate into Tr1 cells by reducing inflammation [41,42]. We suggest that the lack of significant variation of IL-4 levels in pre-therapy *S. mansoni*-infected and co-infected patients is due to the increase in anti-inflammatory Treg and Breg cytokines. 

In relation to immune-regulatory T cell subsets of T reg and B reg responses, IL-10 and IL-35 were studied, respectively. A significant increase in IL-10 in the pre-therapy groups of both *Schistosoma* species is universally associated with mechanisms of inhibition and of negative regulation of both T and B cell cascades [12,43]. The increase in IL-10 may suggest a systemic general downregulation of immunity caused by the parasite and/or its products, as already demonstrated for other helminths [44]. High levels of IL-10 could be stimulated by a high intensity of infection to prevent the development of an excess pathology mediated by Th2 in addition to the Th1-mediated pathology [45]. Our data regarding the *S. mansoni* co-infected patients did not differ from that of single-infected patients, thus discounting any confounding effects for this cytokine in *Schistosoma* mixed infections. Other investigators have also found significantly increased IL-10 levels in humans with *Schistosoma* infections [46], and similar findings have also been demonstrated in other helminth infections, such as *Ascaris lumbricoides* [47]. This is an interesting study area regarding the potential for treating human auto-immune and allergy-related diseases with live helminths or helminth-derived products.

The novel finding of this study was the increased serum level of IL-35, an important anti-inflammatory cytokine secreted by Breg cells [25,26], in *S. haematobium*-infected patients before treatment, compared with the same patient group after treatment. Moreover, IL-35 was found significantly increased during both *S. mansoni* mono-infection and co-infection by *S. mansoni* with other parasites. Eradication of *S. mansoni* after anti-parasitic therapy was associated with reduced IL-35 to concentrations not significantly different from healthy volunteers. Based on these results, IL-35 might play a pivotal role in the Breg immunological cascade and as a prognostic tool to evaluate the therapeutic activity of antiparasitic drugs (antiparasitic drug stewardship). No significant differences in IL-35 were found between single and co-infected *S. mansoni* patients, thereby discounting any confounding factor of co-infection for this cytokine. This general scenario aligns with the balance of the pro- and anti-inflammatory cytokines already reported in other helminth human infections [48].

A limitation of this study could be the low number of patients, predominantly young African males, which reflects most of the immigrant population in Italy in recent years [49].

## 5. Conclusions

In conclusion, the thorough study of pre-therapy and post-therapy profiles of Th1, Th2, Treg, and Breg cytokines revealed a depression of the Th1 and Th2 cascades and an increase in Treg and Breg cytokine concentrations. The immune depression role of IL-10 during parasite diseases is already reported in the literature, although most findings are based on in vitro and in vivo studies. Conversely, the novel feature of the present study is the behavior of the Breg cytokine (IL-35), which plays a further role in the control of the Th1 and Th2 cascades and seemed to be independent of co-infection with other parasites. Therefore, IL-35 may be a candidate for a biomarker role in the evaluation of patient treatment following *Schistosoma* infection.

## Figures and Tables

**Table 1:** Demographic characteristics and microscopic examination data from biological samples of chronic *Schistosoma*-infected patients.

ID Patient	Age	Sex	Country of Origin	Sample Collected	Parasite Identified by Microscopy
1	23	M	Guinea	Urine	*S. haematobium*
2	21	M	Sierra Leone	Urine	*S. haematobium*
3	23	M	Ivory Coast	Urine	*S. haematobium*
4	19	M	Ghana	Urine	*S. haematobium*
5	23	M	Mali	Urine	*S. haematobium*
6	20	M	Cameroon	Urine	*S. haematobium*
7	20	M	Liberia	Urine	*S. haematobium*
8	20	M	Brazil	Urine	*S. haematobium*
9	29	M	Ghana	Urine	*S. haematobium*
10	32	M	Mali	Urine	*S. haematobium*
11	20	F	Mali	Urine	*S. haematobium*
12	21	F	Mali	Urine	*S. haematobium*
13	20	M	Senegal	Urine	*S. haematobium*
14	21	M	Senegal	Urine	*S. haematobium*
15	18	M	Mali	Urine	*S. haematobium*
16	18	M	Guinea	Stool	*S.mansoni*
17	22	M	Senegal	Stool	*S.mansoni*
18	19	M	Senegal	Stool	*S.mansoni*
19	29	F	Ivory Coast	Stool	*S.mansoni*
20	29	M	Camerun	Stool	*S.mansoni*
21	19	M	Liberia	Stool	*S.mansoni*
22	31	M	Ghana	Stool	*S.mansoni*
23	23	M	Ivory Coast	Stool	*S.mansoni*
24	25	M	Gambia	Stool	*S. mansoni*
25	22	M	Bangladesh	Stool	*S. mansoni*
26	21	M	Ivory Coast	Stool	*S. mansoni*
27	19	M	Sierra Leone	Stool	*S. mansoni, E.coli*
28	23	M	Ivory Coast	Stool	*S. mansoni, E.nana*
29	28	M	Guinea	Stool	*S. mansoni, Blastocystis* spp.
30	28	M	Ethiopia	Stool	*S. mansoni, Blastocystis* spp.
31	19	M	Guinea	Stool	*S. mansoni, G.intestinalis*
32	30	M	Ivory Coast	Stool	*S. mansoni, E. nana*
33	24	F	Brazil	Stool	*S. mansoni, E.* *nana* *, E. coli, C.mesnili*
34	19	M	Mali	Stool	*S. mansoni,* *I.butschlii,* *E.* *col* *, E. h/d/m*
35	33	M	Mali	Bladder biopsy *	*Hybrid* *S. haematobium-bovis*

* The *S. haematobium–bovis* hybrid in bladder biopsy was identified by molecular characterization using Sanger sequencing and phylogenetic analysis [6].

## Data Availability

All data were referred in the main text.
